# Virulence gene profiles, biofilm formation, and antimicrobial resistance of *Vibrio cholerae* non-O1/non-O139 bacteria isolated from West Bengal, India

**DOI:** 10.1016/j.heliyon.2018.e01040

**Published:** 2018-12-17

**Authors:** Parimal Dua, Amit Karmakar, Chandradipa Ghosh

**Affiliations:** Microbiology Laboratory, Department of Human Physiology with Community Health, Vidyasagar University, Paschim Medinipur, West Bengal 721102, India

**Keywords:** Microbiology, Infectious disease, Public health

## Abstract

*Vibrio cholerae* is the causative agent of acute dehydrating diarrhoeal disease cholera. Among 71 *V. cholerae* non-O1/non-O139 isolates, all yielded negative results for *ctxA*, *ctxB* and *tcpA* genes in PCR assay. Few strains were positive for *stn* (28.38%), and *ompU* (31.08%) genes. While all isolates were negative for *ace* gene, only two were positive for *zot* gene. All strains expressed *toxR* and *toxT* genes. It was also found that all isolates were slime-producer and these were capable of forming moderate to high biofilm. Biofilm formation was controlled positively by the transcriptional regulators VpsR and VpsT and was regulated negatively by HapR, as well as CRP regulatory complex. These isolates were resistant to ampicillin, furazolidone, doxycycline, vancomycin, erythromycin, while these were susceptible to ciprofloxacin, gentamycin, kanamycin, polymixin B, norfloxacin, chloramphenicol, sulphamethoxazole-trimethoprim, tetracycline, nalidixic acid, and streptomycin. Indeed, 69.01% isolates were resistant to multiple antibiotics (MAR: resistance to 3 or more antibiotics). Treatment protocols for cholera patients should be based on local antibiogram data.

## 1 Introduction

*V. cholerae* is the causative agent of an acute dehydrating diarrhoeal disease cholera that is still endemic in many developing countries. Non-O1/non-O139 serogroups of *V. cholerae* usually cause some cases of mild gastroenteritis (Kaper et al., 1995). Some non-O1/non-O139 *V. cholerae* strains carry significant virulence genes contained in the CTX prophage which encodes CT (Cholera Toxin) and the TCP (Toxin Coregulated Pilus) pathogenicity island encoding the major colonization factor TCP these are usually carried by epidemic *V. cholerae* O1 and O139 strains (Faruque et al., 2003). Some non-O1/non-O139 *V. cholerae* strains may also carry other virulence factors such as heat-stable enterotoxin (*stn*) (Arita et al., 1986; Guglielmetti et al., 1994; Ogawa et al., 1990), outer membrane protein (*omp*U) (Sperandio et al., 1996), a ToxR regulatory protein (Miller et al., 1987) and a zonula occludens toxin (*zot*) (Fasano et al., 1991). Transcriptional regulators including ToxR and ToxT are involved in activating transcription required for coordinate expression of several virulence genes concerned with pathogenicity of *V. cholerae* (Champion et al., 1997). Hence, detection and monitoring of toxigenic *V. cholerae* non-O1/non-O139 are important during surveillance.

During their life cycle both in aquatic environment and eukaryotic host *V. cholerae* face a number of stresses i.e., chlorine water, antibiotics, bactericidal agents etc and to combat these stresses they have evolved an adaptive feature known to be formation of biofilm on biotic and abiotic surfaces. Biofilm formation plays key role in the ecology and transmission of *Vibrio* species. Attached bacteria may form monolayer of cells dispersed on a surface, they may get clustered on surface in microcolonies, or they may be organized into a three-dimensional biofilm (Costerton et al., 1995). Microcolonies are specialized and adapted form of surface growth which is formed by gathering of bacteria that develop three-dimensional tectonics composed of an extracellular polysaccharide (EPS), nucleic acids and proteins produced by resident bacteria (Frolund et al., 1995; Sutherland, 2001). Biofilm is composed of EPS 85% in depth and EPS production is crucial for the development of a mature biofilm (Costerton et al., 1995; Kolter and Losick, 1998). Biofilm are also resistant to the immune defense responses of the host (Jensen et al., 2010; Hänsch, 2012).

Antibiotics are only recommended for the treatment of cholera patients with severe dehydration. The use of antibiotics reduces the volume of dehydration and shortens the duration and severity of diarrhea. It also reduces the transmission of infection to others (Sack et al., 2004). *V. cholerae* develop resistance against many antibiotics which are generally used to treat cholera (Sack et al., 2004). However, the recently isolated *V. cholerae* strains in India have been found to be widely resistant to multiple drugs including ampicilin, streptomycin, ciprofloxacin, chloramphenicol, tetracycline, nalidixic acid, sulfamethoxazole and trimethoprim (Sabu et al., 2007; Kumar et al., 2010). A study undertaken in India has reported these organisms to be resistant to nalidixic acid, ciprofloxacin, co-trimoxazole, chloramphenicol, tetracycline, cephalexin and ampicilin antibiotics (Sabeena et al., 2001; Kingston et al., 2009). Furthermore, another study also found fluoroquinolone and tetracycline resistance to increase in the clinical isolates of *V. cholerae* in India (Garg et al., 2001; Roychowdhury et al., 2008).

The aim of our study was to examine the *V. cholerae* non-O1/non-O139 strains in detail to obtain an understanding of the virulence traits, antibiotic resistance pattern, biofilm formation includes its regulation which might have contributed to the pathogenesis of the isolates.

## 2 Materials and methods

### 2.1 Samples and ethical approval

A total of 78 clinical strains of *V. cholerae* were isolated from 147 stool samples of the diarrheal patients admitted in hospital at Paschim Medinipore in West Bengal, India in the year 2013 according to WHO method (WHO, 1987). A detail about this was mentioned in our previous study (Dua et al., 2017). Our protocol was approved by the Institutional Ethics Committee (IEC) of Vidyasagar University (IEC/8-1/C-1/17). Women included in the study were diagnosed to have Cholera. A written informed consent was obtained from each patient before inclusion in the study.

### 2.2 PCR amplification

The PCR was performed as described by Chun et al. (1999) in order to determine the presence of toxin genes. DNA from *V. cholerae* non-O1/non-O139 strains used for the PCR template was prepared from overnight LB-broth cultures at 37 °C. The culture was centrifuged at 10,000 g for 5 min and the pellet was suspended in 1ml sterile Milli Q water (Millipore-Synergy®, USA). The suspension was boiled for 10 min. and the boiled suspension was centrifuged at 12,000 g for 5 min. After centrifugation the supernatant was stored at −20 °C (Adabi et al., 2011). Primers used in this study for the detection of selected virulence and regulatory genes in *V. cholerae* were *ctxA, ctxB, tcpA* (classical & El Tor), *ace, zot, stn, toxR, toxT, vpsR, crp,* and *hapR* genes. A more comprehensive list of relevant targets, with PCR conditions and expected amplicons, are listed in Table 1. PCR was carried out in 20μl volumes containing 2μl template DNA, 10μl PCR master mixture containing 2μl 10x concentrated PCR buffer [100 mM Tris/HCl, (pH 8.3), 500 mM KCl], 1.2 μl 15 mM MgCl_2_, 2 μl dNTPs mixture (2.5 mM each dNTP), 0.5 μl (5 U μl-1) Taq DNA polymerase and 4.3 μl sterilized Millipore distilled water and 4 μl (5 pmol μl-1) each of appropriate primers. All PCR assays were performed using an automated thermal cycler (Ependroff, Germany).

**Table 1:** Details of PCR primers, PCR conditions and amplicon sizes used in this study for the detection of virulence and regulatory genes.

Target gene	Direction	Primer sequence (5ʹ- 3ʹ)	Amplicon size (bp)	PCR condition			Reference
				Denaturation	Annealing	Extension	
***ctxA***	F	CTC AGA CGG GAT TTG TTA GGC ACG	301	94 °C 1 min	55 °C, 45 sec	72 °C 1 min	(Shirai et al., 1991)
	R	TCT ATC TCT GTA GCC CCT ATT ACG					
***ctxB***	F	GGT TGC TTC TCA TCA TCG AAC CAC	460	94 °C 1 min	58 °C, 1 min	72 °C 1 min	(Olsvik et al., 1993)
	R	GAT ACA CAT AAT AGA ATT AAG GAT					
***tcpA-*class**	F	CAC GAT AAG AAA ACC GGT CAA GAG	620	94 °C 1 min	58 °C, 45 sec	72 °C 1 min	(Rivera et al., 2001)
	R	TTA CCA AAT GCA ACG CCG AAT G -3′					
***tcpA*-El Tor**	F	CAC GAT AAG AAA ACC GGT CAA GAG	453	94 °C 1 min	55 °C, 45 sec	72 °C 1 min	(Rivera et al., 2001)
	R	CGA AAG CAC CTT CTT TCA CAC GTT G					
***stn***	F	GAG AAA CCT ATT CAT TGC	216	94 °C 1 min	54 °C, 30 sec	72 °C 45 sec	(Vicente et al., 1997)
	R	GCA AGC TGG ATT GCA AC					
***Zot***	F	TCG CTT AAC GAT GGC GCG TTT T	947	94 °C 1 min	58 °C, 45 sec	72 °C 1 min	(Rivera et al., 2001)
	R	AAC CCC GTT TCA CTT CTA CCC A					
***ompU***	F	ACG CTG ACG GAA TCA ACC AAA G	869	94 °C 1 min	55 °C, 1 min	72 °C 1 min	(Rivera et al., 2001)
	R	GCG GAA GTT TGG CTT GAA GTA G					
***ace***	F	TAA GGA TGT GCT TAT GAT GGA CAC CC	316	94 °C 1 min	55 °C, 30 sec	72 °C 45 sec	(Shi et al., 1998)
	R	CGT GAT GAA TAA AGA TAC TCA TAG G					
***toxR***	F	CCT TCG ATC CCC TAA GCA ATA C	779	94 °C 1 min	58 °C, 1 min	72 °C 1 min	(Rivera et al., 2001)
	R	AGG GTT AGC AAC GAT GCG TAA G					
***toxT***	F	TTG CTT GGT TAG TTA TGA GAT	581	94 °C 1 min	56 °C, 45 sec	72 °C 1 min	(Kondo and Ajawatanawong, 2009)
	R	TTG CAA ACC CAG ACT GAT AT					
***vpsR***	F	TAGAGCACGGCTTACCGCCA	649	94 °C 1 min	63 °C 1 min	72 °C 1 min	This study
	R	GCCAGCCAACGGACTTGCTT					
***crp***	F	CGCGGGTGAGAAAGCGGAAA	286	94 °C 1 min	63 °C 1 min	72 °C 1 min	This study
	R	CACTTGCAGACGACGAGCCA					
***hapR***	F	GGTACTATACGCGCCACCAA	191	94 °C 1 min	60 °C 1 min	72 °C 1 min	This study
	R	GAACCACGCAGCAATCCAAC					

F, forward; R, Reverse.

### 2.3 Gel electrophoresis

The amplified products were then separated by agarose gel electrophoresis. PCR-products were run on 1% agarose gels (HiMedia, Mumbai, India) containing Ethidium Bromide stain (EtBr) (HiMedia, Mumbai, India) with 1x TAE buffer (40 mM Tris- HCl, 20 mM Naacetate, 1mM EDTA, pH 8.4) and the bands were visualized under an UV transilluminator (Biometra, Germany). Images were captured with digital imaging system (Bio-Rad).

### 2.4 Slime production assay

Qualitative detection of biofilm formation was studied by culturing the isolated strains under study on Congo red agar (CRA) plates (Freeman et al., 1989). CRA plates were prepared by mixing 0.8 g Congo red with 36 g saccharose (Himedia, Mumbai) in 1 L of brain heart infusion agar. *V. cholerae* non-O1/non-O139 strains were inoculated into the surface of CRA plates, and were incubated for 24 h at 30 °C under aerobic conditions and followed overnight at room temperature (Chaieb et al., 2007). Slime producing bacteria appeared as black colonies, whereas non-slime producers remained non pigmented (Subashkumar et al., 2006).

### 2.5 Biofilm assay

Biofilm formation ability of *V. cholerae* non-O1/non-O139 strains was quantified by an assay method using crystal violet (CV) staining (Lauriano et al., 2004). For the biofilm formation, cells were grown in LB broth at 30 °C under static condition in borosilicate glass tubes. Following 22 hours of incubation, the cultures were removed, and the tubes were washed twice gently with distilled water to remove loosely bound cells from the surface. Adherent cells were then stained with 1% crystal violet (w/v in distilled water) solution and after 10 minutes, the dye solution was removed and washed three times thoroughly with distilled water and treated with dimethyl sulfoxide (DMSO). Biofilm formation was investigated using *V. cholerae* O139 Bengal strain MO10 as a reference strain which showed robust biofilm formation ability under the test conditions. Biofilm formation was measured photometrically at OD 570 nm, (Spectrophotometer, Schimadzu, Japan) (Joelsson et al., 2006). Indeed, the optical density value (OD) of 0.480 was the mean OD of three OD of biofilm activity of Blank and the OD of 0.800 was the mean OD of three OD of biofilm activity of *V. cholerae* O139 (MO10). The biofilm formation was defined as none (<0.480), weak/intermediate (0.480–0.800) and strong (>0.800). Strains that showed strong or intermediate biofilm formation were rated as positive. The results also showed that isolated *V. cholerae* non-O1/non-O139 strains were able to produce biofilm on abiotic surface.

### 2.6 Antimicrobial susceptibility

Antimicrobial susceptibility analysis of *V. cholerae* non-O1/non-O139 strains was performed by Disk diffusion method on Muller Hinton agar using antibiotic Disk obtained from HiMedia, Mumbai (Bauer et al., 1966). The antibiotics were ampicilin (A, 10μg), chloramphenicol (C, 30μg), polymixin-B (PB, 50μg), streptomycin (S, 30μg), nalidixic acid (Na, 30μg), tetracycline (T, 30μg), erythromycin (E, 15μg), kanamycin (K, 30μg), vancomycin (V, 15μg), sulfamethoxazole-trimethoprim (SXT, 25 μg), gentamicin (G, 10μg), furazolidone (F-50μg), norfloxacin (Nx-10μg), ciprofloxacin (Cf, 5μg) and doxycycline (Do, 10μg). The isolates scored as sensitive or resistant as per the CLSI guidelines (CLSI, 2015).

A model *V. cholerae* strain used for the preset study was *V. cholerae* O139 Bengal strain MO10, the strain with high epidemiological importance and representative strain for the group of *V. cholerae* organisms in which slime production, biofilm formation, antimicrobial susceptibility and expression of virulence factors has been identified.

## 3 Results and discussion

Seventy one *V. cholerae* non-O1/non-O139 strains used in this study were isolated from diarrheal patients during 2013 in Paschim Medinipur, West Bengal, India already mentioned in our previous report (Dua et al., 2017).

### 3.1 Virulence gene profiles

The *V. cholerae* non-O1/non-O139 strains were characterized in detail to obtain an understanding of the role of the virulence traits in cholera-like diarrhoea. The *V. cholerae* non-O1/non-O139 strains were screened for the presence of different virulence genes like *ctxA*, *ctxB*, *tcpA* (classical & El Tor), *zot*, *st, ace*, *ompU* which are found significant for pathogenesis. It has been shown that all the isolated strains yielded negative results for *ctxA*, *ctxB* and *tcpA* (classical & El Tor) genes in PCR assay. Furthermore, in the present study few strains of *V. cholerae* non-O1/non-O139 were positive by PCR for *st* (28.38%), *zot* (2.70%) and *ompU* (31.08%) genes by PCR which play some role in the disease process (Arita et al., 1986; Bagchi et al., 1993). In another study, outer membrane protein (OmpU) was reported to be a potential adherence factor for *V. cholerae* bacteria (Sperandio et al., 1996). Further analysis on *V. cholerae* non-O1/non-O139 strains showed that all strains were negative for *ace* gene. In an earlier study, it was shown that *V. cholerae* non-O1/non-O139 isolates including clinical sources were negative for *zot* gene (Rivera et al., 2001). Although an earlier study reported isolates of diarrheal outbreaks from Chennai to be positive for virulence and regulatory genes *ctxA*, *tcpA*, *ace*, *zot, ompU* and *toxR* (Kingston et al., 2009). Further analysis of these *V. cholerae* non-O1/non-O139 strains revealed all strains to express *toxR* and *toxT* genes, encoding the transcriptional regulators ToxR and ToxT respectively generally present in *V. cholerae* strains (Bidinost et al., 2004). One study showed that all of the isolates of *V. cholerae* non-O1/non-O139 (except VO22) were positive for the gene encoding the central regulatory protein, ToxR (Singh et al., 2001). In another study, it was also shown that all of 13 isolates (100%) of *V. cholerae* non-O1/non-O139 possessed *toxR* and *toxT* genes (Sharma et al., 1998). The *toxR* gene encodes a transcriptional activator controlling CT gene expression, TCP biogenesis, outer membrane protein expression and at least 17 distinct genes in *V. cholerae* O1 and *V. cholerae* O139 strains (DiRita, 1992; Herrington et al., 1988; Miller et al., 1987). One former study revealed that all the *V. cholerae* non-O1/non-O139 strains isolated from Kolkata regions of West Bengal in 2003 possessed *toxR*, the central regulatory protein gene (Singh et al., 2001). According to Rivera et al. (2001) all of the *V. cholerae* non-O1/non-O139 strains studied, regardless of whether they were toxigenic or non-toxigenic, were found to possess the *toxR,* regulatory gene. The presence of *toxR* (100%) and *toxT* (100%) genes in the *V. cholerae* non-O1/non-O139 strains suggest that they are required for the functioning of the organism and are not only related to pathogenesis (Rivera et al., 2001). This study screened for the presence of *tcpA* gene in the *V. cholerae* isolates by PCR but negative result was obtained. Previous reports have shown that *V. cholerae* non-O1/non-O139 strains isolated from clinical sources rarely possess the *tcpA* and the CTX genetic elements (Mukhopadhyay et al., 1996; Sharma et al., 1998). The negative results in PCR for the following genes like *ctxA*, *ctxB*, *zot, tcpA*, *st, ace, ompU* suggest that these genes are absent or may be due to non-amplification of the primer binding region in those genes or it may be due to sequence divergence in primer binding site. In *V. cholerae* non-O1/non-O139 isolates, the genotypes were diverse. From the results of this study, it is postulated that in the absence of major virulence factors, *V. cholerae* non-O1/non-O139 strains isolated from hospitalized patients have the ability to cause diarrhea by a mechanism entirely different from that of the toxigenic *V. cholerae* O1 and O139 strains. Furthermore, the isolated *V. cholerae* non-O1/non-O139 strains are potentially less virulent than the conventional *V. cholerae* O1 and O139 strains. As the isolates of *V. cholerae* non-O1/non-O139 are associated with sporadic infections (Sharma et al., 1998), these strains can no longer be ignored. Furthermore, it is noteworthy that *V. cholerae* non-O1/non-O139 strains have been reported to be involved in the emergence of a newer variant of *V. cholerae* and the fact is supported by the genesis of *V. cholerae* O139, which is believed to have evolved as a result of horizontal gene transfer between the O1 and the non-O1 serogroups (Bik et al., 1995). In addition, the possible conversion of *V. cholerae* from non-O1 to O1 serotype has provided added interest (Colwell et al., 1995).

### 3.2 Slime production of *V. cholerae*

In our study, it was observed that all *V. cholerae* non-O1/non-O139 strains isolated from clinical samples of Paschim Medinipur were slime-producers. Actually slime production play an important role in the pathogenesis of infections caused by different microorganisms (Alcaráz et al., 2003) and it is also considered to be a significant virulence factor for some staphylococci (Mack et al., 2000) as well as for *Aeromonas* spp. which indicates the high risk source contamination (Sechi et al., 2000). Although slime is generally composed with polysaccharide but other polymers may also be present and they are probably involved in the protection of microbial cells. In addition, *V. cholerae* which produce these exopolymers are more resistant to desiccation, predation and toxic chemicals (Ophir and Gutnick, 1994). However, these molecules also play significant role in the formation of biofilms on solid surfaces. Therefore, exopolymers were considered to be associated in the initial steps of biofilm formation (Muller et al., 1993).

### 3.3 Biofilm formation

**Fig. 1:**
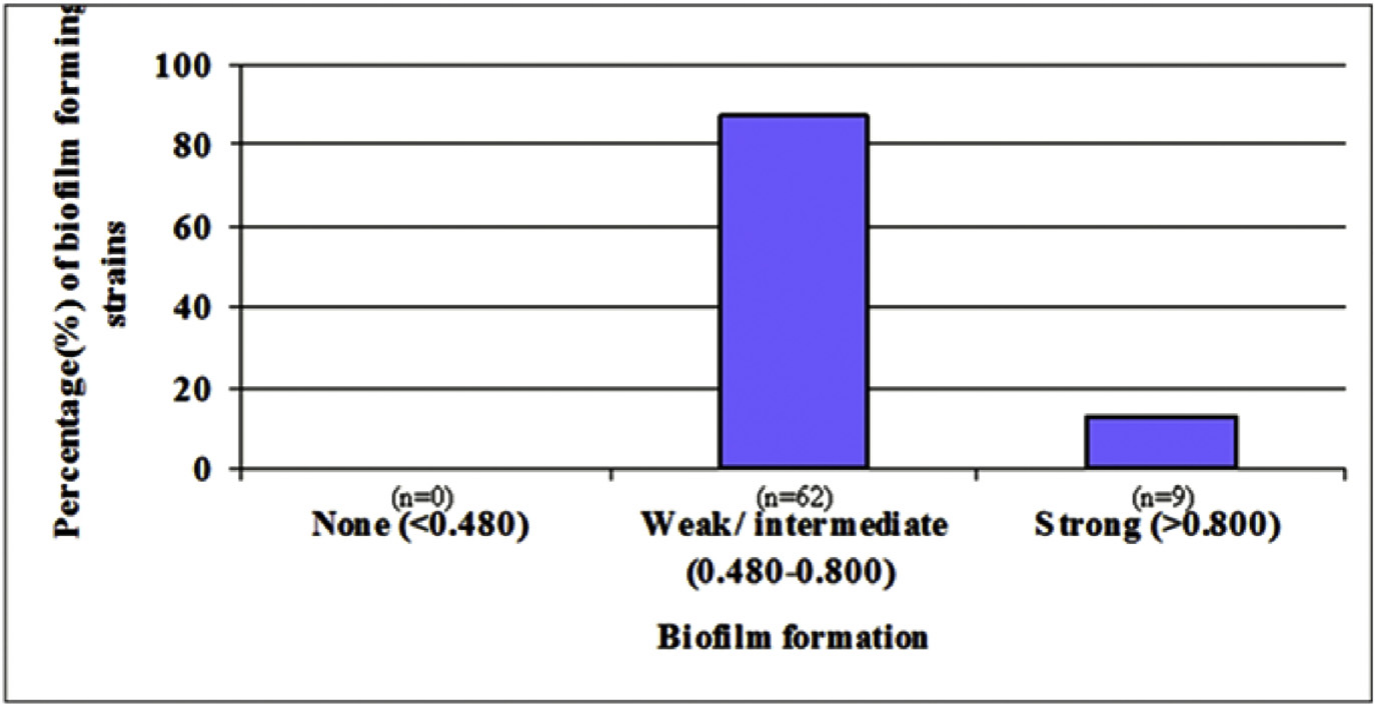
Biofilm formation ability of *V. cholerae* non-O1/non-O139 strains isolated from clinical samples of Paschim Medinipur.

Qualitative adherence of isolated *V. cholerae* non-O1/non-O139 strains performed on glass test tube and it was found that most of *V. cholerae* non-O1/non-O139 strains were highly adherent. In our study it was found that among 71 tested strains, all *V. cholerae* non-O1/non-O139 strains were capable of forming biofilm (Fig. 1). We observed that the majority of the *V. cholerae* non-O1/non-O139 strains were able to produce moderate to high biofim (Fig. 1). Indeed, biofilm formation gets initiated with the attachment of bacteria to abiotic surfaces by pili, flagella or other structures and followed by the production of exopolysaccharides to form a glycocalyx (Wong et al., 2002).

The stability of biofilm structure is critically determined by expression of exopolysaccharide (EPS). Vibriopolysaccharide (VPS) is essential for the development of three-dimensional biofilm structures. The polysaccharide gets secreted from cell surfaces shortly after initial attachment and VPS extrusion from cells was observed throughout biofilm development. VpsR was the master regulator of biofilm formation in *V. cholerae.* The expression of VpsR was positively regulated by VpsT and negatively regulated by HapR. HapR is the primary negative regulator of biofilm formation in *V. cholerae*. The global regulator cyclic AMP receptor protein (CRP) has been shown to upregulate HapR production. The second messenger cAMP has been identified to be involved with various cellular responses and acts as a repressor of *V. cholerae* biofilm formation.

### 3.4 Antibiotic susceptibility of *V. cholerae*

**Fig. 2:**
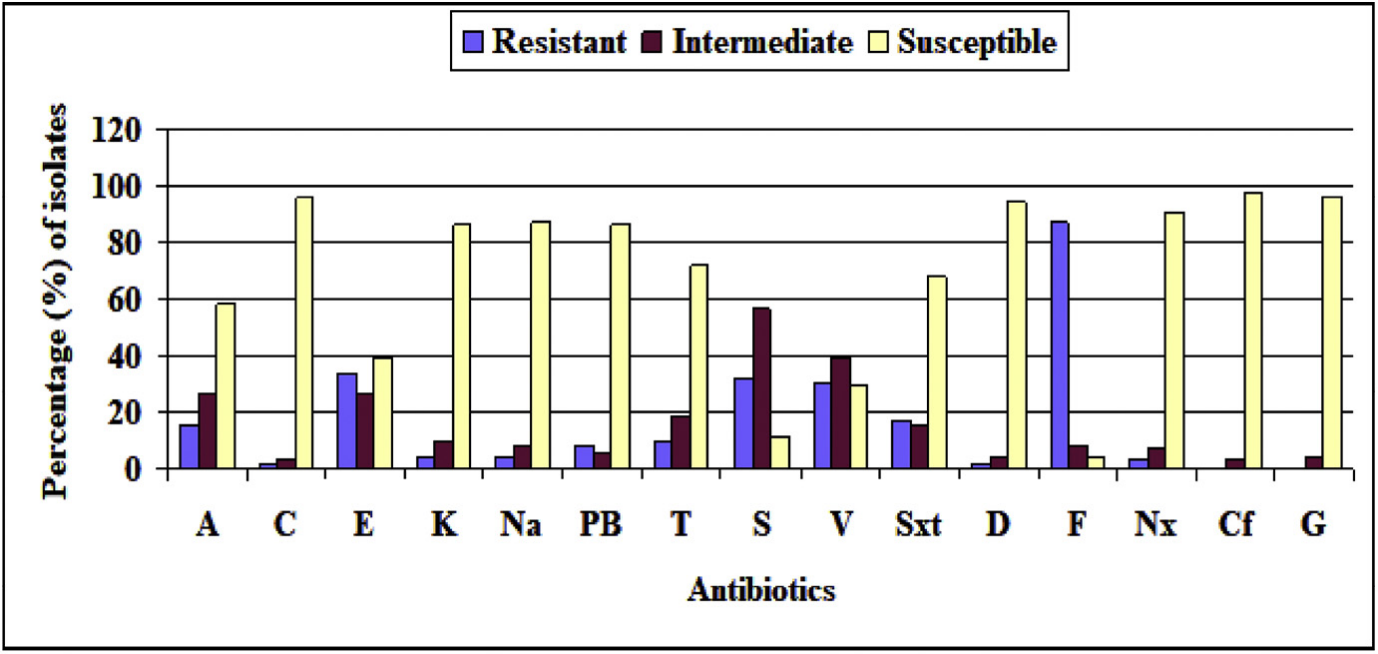
Antibiotic susceptibilities of *V. cholerae* non-O1/non-O139 strains isolated from clinical sources of Paschim Medinipur; A; Amplicilin, C; Chloramphenicol, E; Erythromycin, K; Kanamycin, Na; Nalidixic acid, PB; Polymixin B, T; Tetracycline, S; Streptomycin, V; Vancomycin, Sxt; Sulphamethoxazole-trimethoprim, D; Doxycycline, F; Furazolidone, Nx; Norfloxacin, Cf; Ciprofloxacin, G; Getamycin.

Generally, standard rehydration therapy has been used for diarrhoeal patients. Indeed, standard rehydration therapy alone can reduce cholera mortality, but it has not been shown to reduce the duration of illness (Guerrant et al., 2003). Therefore, antimicrobial therapy has been shown to reduce the magnitude of fluid loss, duration of illness and duration of excretion. The antibiotic resistance profile of all the *V. cholerae* non-O1/non-O139 strains (71) against 15 different antibiotics has been presented in Fig. 2. Susceptibility or resistance to the antibiotics was ascertained as per the guidelines of CLSI (CLSI, 2015). The clinical isolates of Paschim Medinipur were resistant to ampicillin (100%), furazolidone (95.77%), doxycycline (88.74%), vancomycin (70.43%), erythromycin (60.56%), streptomycin (43.66%), nalidixic acid (42.25%), tetracycline (40.84%), sulphamethoxazole-trimethoprim (32.39%) and chloramphenicol (29.58%) while these isolates were susceptible to ciprofloxacin (97.18%), gentamycin (95.77%), kanamycin (85.92%), polymixin B (85.92%), norfloxacin (80.28%), chloramphenicol (70.42%), Sulphamethoxazole-trimethoprim (67.61%), tetracycline (59.15%), nalidixic acid (57.75%), streptomycin (56.34%) and erythromycin (39.44%) (Fig. 2). In this study the *V. cholerae* non-O1/non-O139 strains showed variable antibiograms. Although isolates from Paschim Medinipur only showed resistance towards doxycycline (Fig. 2), a prior report demostrated that doxycycline was effective against majority of the *V. cholerae* strains (Kingston et al., 2009). Ciprofloxacin resistance for the El Tor strains has also been reported elsewhere (Garg et al., 2000; Dalsgaard et al., 1999) although *V. cholerae* non-O1/non-O139 strains recovered from Paschim Medinipur were sensitive to ciprofloxacin. This result is consistent with the result of the El Tor strains as well as the O139 strains isolated from the Chennai of India during the year 2002–2004 (Kingston et al., 2009). In addition, gentamycin resistance has been reported against majority of the *V. cholerae* strains elsewhere (Kingston et al., 2009) but all the *V. cholerae* non-O1/non-O139 recovered from Paschim Medinipur were gentamycin sensitive. The antibiotic resistance pattern which was found in this study is consistent with a previous report including *V. cholerae* non-O1/non-O139 strains those were isolated in Kolkata, India (Chatterjee et al., 2009). In our study, it has been found that 69.01% *V. cholerae* non-O1/non-O139 strains were resistant to multiple antibiotics (MAR: resistance to 3 or more antibiotics), those varied among the individual isolates. In addition, 30.99% *V. cholerae* non-O1/non-O139 strains exhibited the highest resistance (resistant to about 8 of the 15 antibiotics tested). Antimicrobial susceptibility has seen with wide variation in the isolated *V. cholerae* non-O1/non-O139 strains during our study period. The antibiotic resistance of *V. cholerae* strains that have been isolated from diarrhoeal patients has increased.

From the results of this study, it is concluded that *V. cholerae* non-O1/non-O139 strains can no longer be ignored. Because of increasing antibiotic resistance, antibiotic susceptibility testing reports on *V. cholerae* strains isolated from patients may be helpful for antimicrobial therapy in severe infections. Results of antibiogram suggest that multidrug resistance is prevalent in the isolates of *V. cholerae* non-O1/non-O139 in Paschim Medinipur, West Bengal, India. Therefore, the genetic elements associated with virulence and drug resistance in *V. cholerae* non-O1/non-O139 strains are diverse. The antibiotic resistance profile may be due to loss or acquisition of genetic material which are responsible for drug resistance. So it becomes difficult to control the disease with common antimicrobial therapy. However, because of increasing global antimicrobial resistance, commonly used antibiotics are no longer recommended as first-line therapy. Whenever possible, treatment protocols for cholera patients should be based on local antibiogram data.

## Declarations

### Author contribution statement

Parimal Dua: Conceived and designed the experiments; Performed the experiments; Analyzed and interpreted the data; Contributed reagents, materials, analysis tools or data; Wrote the paper.

Amit Karmakar, Chandradipa Ghosh: Performed the experiments; Analyzed and interpreted the data; Contributed reagents, materials, analysis tools or data.

### Funding statement

This research did not receive any specific grant from funding agencies in the public, commercial, or not-for-profit sectors.

### Competing interest statement

The authors declare no conflict of interest.

### Additional information

No additional information is available for this paper.
